# Effect of 12 Weeks High Oleic Peanut Consumption on Cardio-Metabolic Risk Factors and Body Composition

**DOI:** 10.3390/nu7095343

**Published:** 2015-09-02

**Authors:** Jayne A. Barbour, Peter R. C. Howe, Jonathan D. Buckley, Janet Bryan, Alison M. Coates

**Affiliations:** 1 Alliance for Research in Exercise, Nutrition and Activity, University of South Australia, G.P.O. Box 2471, Adelaide, South Australia 5001, Australia; E-Mails: Jayne.barbour@unisa.edu.au (J.A.B.); Jon.buckley@unisa.edu.au (J.D.B.); 2 Clinical Nutrition Research Centre, University of Newcastle (PRCH), University Drive Callaghan, New South Wales 2308, Australia; E-Mail: Peter.howe@newcastle.edu.au; 3 Department of Psychology, Social Work and Social Policy, University of South Australia, G.P.O. Box 2471, Adelaide, South Australia 5001, Australia; E-Mail: Janet.bryan@unisa.edu.au

**Keywords:** high-oleic peanuts, inflammation, lipids, glucose, insulin, body weight

## Abstract

Epidemiological evidence indicates an inverse association between nut consumption and obesity, inflammation, hyperlipidaemia and glucose intolerance. We investigated effects of high oleic peanut consumption *vs.* a nut free diet on adiposity and cardio-metabolic risk markers. In a randomised cross-over design, 61 healthy subjects (65 ± 7 years, body mass index (BMI) 31 ± 4 kg/m^2^) alternated either high oleic peanuts (15%–20% of energy) or a nut free diet for 12 weeks. Body composition and mass, waist circumference, *C*-reactive protein (CRP), lipids, glucose and insulin were assessed at baseline and after each phase. Repeated measures analysis of variance (ANOVA) compared the two diets. Consistent with other nut studies, there were no differences in lipids, CRP, glucose and insulin with peanut consumption. In contrast, some reports have demonstrated benefits, likely due to differences in the study cohort. Energy intake was 10% higher (853 kJ, *p* < 0.05), following peanut consumption *vs.* control, attributed to a 30% increase in fat intake (*p* < 0.001), predominantly monounsaturated (increase 22 g, *p* < 0.05). Despite greater energy intake during the peanut phase, there were no differences in body composition, and less than predicted increase (0.5 kg) in body weight for this additional energy intake, possibly due to incomplete nutrient absorption and energy utilisation.

## 1. Introduction

Nuts are rich sources of bioactive nutrients with potential to deliver metabolic and cardiovascular health benefits [1]. Despite peanuts being a legume they share similar nutritional properties to other nuts. Peanuts are an excellent source of protein (approximately 25% of energy) and dietary fibre providing 5%–10% of daily fibre requirements in one serving (30 g), with potential satiety benefits for weight control [2]. Other bioactive nutrients in peanuts such as vitamin E and polyphenols may benefit glucose regulation [3] and inflammation [4,5]. High oleic peanuts are also rich in phytosterols and monounsaturated fat, (providing up to 80% of the fatty acid composition) [6] and they have demonstrated lipid lowering effects [7]. In addition, high oleic peanuts oxidise less readily than regular, higher polyunsaturated peanut varieties (~28% polyunsaturated and 50% monounsaturated fatty acids) hence, have a longer shelf life and are the predominant type of peanut grown in Australia [6].

Evidence suggests that both tree nut and peanut consumption is associated with lower body weight [8]. Several large, cohort studies (the Adventist Health Study, the Iowa Womens’ Health Study and the Physicians Health Study) have shown significant inverse associations between the frequency of nut consumption and body mass index (BMI) [9,10,11] Frequent nut consumption was also associated with reduced risk of weight gain in the SUN (Seguimiento University of Navarra) cohort after 6 years [12]. In the Nurses’ Health Study II, participants who consumed nuts frequently (two or more times per week) had a 31% reduced risk of weight gain, or a 33% lower risk of obesity [13] than those who rarely or never consumed nuts.

Similarly, evidence from intervention studies indicates that incorporating nuts into the diet has little impact on body mass and body fat [14]. A systematic search of 23 clinical trials investigating the effect of chronic nut consumption (averaging ~15%–20% of energy requirements) on body mass demonstrated a small non-significant weighted mean decrease in body weight of 0.47 kg, BMI of 0.40 kg/m^2^, and waist circumference of 1.25 cm [14]; two of these studies assessed peanut intake [15,16]. However, in most of the studies, nuts were used in iso-energetic diets so weight changes were not expected.

Several mechanisms outlined below have been proposed for the lack of weight gain observed with nut consumption, despite their high energy and fat content. These include reduced energy intake subsequent to increased satiety [2], energy lost through faecal fat loss and a possible increase in energy expenditure [8,17].

Nut consumption has also been associated with a reduced risk of type 2 diabetes; evidence to support this comes from large epidemiological studies [18,19]. The Nurses’ Health Study demonstrated that consumption of nuts (~ 5 times per week), peanut butter (~5 times per week) or walnuts (~twice per week) was associated with a 24%, 21% and 15% lower risk respectively of developing type 2 diabetes compared with those who never or rarely ate nuts; the effect was greatest in those of healthy body weight [18]. In addition, the Shanghai Women’s Health Study demonstrated that walnut consumption was associated with a 21% decreased risk of type 2 diabetes [20]. Nut consumption has also improved glycaemic control and insulin sensitivity [21,22]. A literature review [23] revealed 14% weighted mean reductions in fasting insulin/glucose regulation and 34% reduction in homeostatic model assessment (HOMA) scores with nut consumption. However, the effects of nuts on insulin sensitivity are influenced strongly by changes in body weight; this may have accounted for the changes observed in one of the studies where subjects reduced body weight with nut consumption.

Clinical and epidemiological evidence also indicate that nut consumption can enable improvements in inflammatory markers, with daily doses of 30 g able to confer benefits [24,25]. Mediterranean diets in which walnuts [26], mixed nuts [27] or pistachios [22] replaced olive oil have demonstrated improvements in one or more of the inflammatory markers *C-*reactive protein (CRP), intercellular adhesion molecule-1 (ICAM-1), vascular cell adhesion molecule-1 (VCAM-1), and interleukin-6 (IL-6). Weighted mean reductions in ICAM-1 (9%), VCAM-1 (6%) and CRP (12%) were found from analysis of 27 intervention studies with nuts in a literature review [23]. Studies which showed no benefits may have used an insufficient intake of nuts or intervention period.

Clinical trials have clearly shown that intakes of several varieties of nuts can lower total and low density lipoprotein (LDL) cholesterol by 9%–16%, even in the context of healthy diets [28,29,30,31]. A pooled analysis of influences of nut consumption on blood lipids revealed the cholesterol lowering effects are greatest in individuals with higher baseline LDL levels or lower BMI [31]. This pooled analysis estimated that a mean daily consumption of 67 g of nuts reduced LDL by 7%, triglycerides by 5% and reduced LDL:high density lipoprotein (HDL) by 8% [32]. Nut consumption was also found to lower triglyceride levels, primarily in individuals with hypertriglyceridemia. Similarly, a walnut meta-analysis [32] found that walnut-enriched diets significantly decreased total and LDL cholesterol by 5% and 7% respectively with a 5% reduction in triglycerides and no effect on HDL. These lipid lowering effects of nuts appear to be greatest when their intake is substituted for saturated fat in the diet rather than being added to the diet [31].

The cholesterol lowering, anti-inflammatory and glucose regulating benefits of nuts have been attributed to their high content of unsaturated fat, possible weight reduction effect and bioactive compounds, including plant sterols, dietary fibre and antioxidants [30]. Whilst previous studies have investigated the effect of peanut intake on body weight [15,33], few have measured cardio-metabolic outcomes of peanuts and no research has previously been conducted with Australian high oleic peanuts.

This study aimed to investigate the effect of adding high oleic peanuts to habitual diets of healthy overweight adults on cardio-metabolic measures (glucose, insulin, CRP and lipids), body composition and anthropometric measures and to determine if there are any relationships between these outcomes.

## 2. Subjects and Methods

### 2.1. Subjects

Subjects were recruited through Alliance for Research in Exercise, Nutrition and Activity (ARENA) participant data base, newspaper advertisements, flyers and word of mouth. The inclusion criteria were healthy overweight males or post-menopausal females aged between 50 and 75 years with self-reported stable weight and BMI ≥ 25 kg/m^2^. Smokers, regular nut consumers and people with cardiovascular disease, hypertension (>160/100 mmHg), a thyroid condition or nut allergy or those consuming ≥40 g of alcohol/day were excluded. Restrained eaters (determined by a score of ≥12 for the Three Factor Eating questionnaire [34]) were excluded from the study because investigators were interested in how peanuts would be incorporated in the diet by non-dieters who would not be consciously modifying their intake of other foods. This paper reports secondary analysis of data from a study [35] (submitted but not yet published) which was designed to assess a primary cerebrovascular outcome. Sixty four subjects were required to provide 80% power to detect a medium effect size (0.5) in cerebral vascular reactivity. This study was also sufficiently powered to detect a 5% change in body weight, 10% change in LDL and glucose and a 15% change in CRP. Seventy five subjects were recruited to allow for dropouts. The study was approved by the University of South Australia ethics committee (0000026851) and was registered with the Australian and New Zealand clinical trials register (ACTRN 12612000192886).

### 2.2. Study Protocol

In a randomised controlled cross-over trial, subjects attended the research centre on visit one after a 12 h overnight fast for screening; if successful, subjects provided informed consent to commence the study during the same visit. Following baseline measurements of BMI and waist circumference, blood samples were taken by venepuncture. Subjects were randomised using computer generated software to commence either their habitual diet (devoid of nuts) or 12 weeks consumption of high oleic peanuts for 6 out of 7 days each week. Subjects were provided with 4 day physical activity diaries [36] and weighed food diaries along with instructions on how to weigh (to the nearest gram), measure and record intake. These were completed either prior to commencing peanut intake or from the following day (control phase). A second set of diaries was also provided for completion 4 days prior to their next visit. Subjects were reminded not to consume any nuts other than those provided by the study; no other dietary intervention advice was provided. Data entry and analysis was blinded to minimise investigator bias.

At week 6, subjects attended the research centre for measurements of body mass waist circumference and were provided with a further set of diaries to complete prior to the next visit. At week 12 measurements were taken of body composition using dual-energy X-ray absorptiometry (DEXA), body mass, waist circumference and blood samples were taken. Subjects were provided with a further set of diaries to complete 4 days prior to the next visit and then crossed over to the to the alternate treatment arm with repeat visits at weeks 18 and 24.

### 2.3. Test Product

Roasted, unsalted high oleic (Australian “Hi-oleic”, cultivar Middleton) peanuts with skins on were provided in 28 g sealed foil packets by the Peanut Company of Australia (Kingaroy, Queensland, Australia); their profile is shown in Table 1. Males were provided with 3 packets (84 g) per day and females 2 packets (56 g) per day to be consumed for 6 days per week (providing ~15%–20% of energy intake). To ensure maximum nutrients were obtained from the peanuts, subjects were asked to consume any skins which had fallen off in the packaging. To assist with compliance subjects were given one rest day per week with no peanuts.

**Table 1:** Nutrient content of high oleic peanuts.

Nutrient	Amount Per 100 g *	Nutrient	Amount Per 100 g
Energy (kJ)	2376	Fibre (g)	8.5
MUFA (oleic) (g)	38	Vitamin E (mg)	8.3
PUFA (linoleic) (g)	2	Folate (μg)	240
SFA (palmitic) (g)	3	Magnesium (mg)	168
Protein (g)	26	Potassium (mg)	705
Arginine (g)	3	Iron (mg)	4.6
Resveratrol (mg)	0.2	Zinc (mg)	3.3

Abbreviations: MUFA, monounsaturated fatty acids; PUFA, polyunsaturated fatty acids; SFA, saturated fatty acids; * Peanut Company of Australia [6].

### 2.4. Anthropometric Measures

Weight was measured to the nearest 200 g using an electronic scale (Tanita Ultimate Scale 2000; Tokyo, Japan) with subjects wearing light clothing and without shoes. Two measurements were taken with the average used. If measures differed by more than 1% a 3rd measure was taken. Height was measured to the nearest 0.1 cm using a wall-mounted telescopic stadiometer (Seca220; Vogel & Halke, Hamburg, Germany) with subjects in stockinged or bare feet and two measurements taken and the average used. Waist circumference was measured with a metal anthropometric tape measure (Lufkin W606PM, Cooper Tools, Apex, North Carolina, USA) at the natural waist or narrowest part of the torso to the nearest 0.1 cm with two measurements taken and the average used.

### 2.5. Body Composition

Body composition measuring total body fat mass, percentage body fat and lean tissue mass was performed by DEXA (Luna Corp Prodigy Advance^®^ Model GE, Madison, WI, USA). Subjects were required to remove all items of jewelry, over clothes and under garments with metal fixings and any surgical devices or prosthesis were noted. The machine was calibrated prior to each scan with details of subjects’ body weight, height and date of birth entered. Subjects were scanned supine, positioned centrally on the mat and restrained at the ankle to minimise movement. DEXA is limited to subjects under 135 kg hence those over this weight were excluded. Previous reports from our research centre have found the standard error of the measurement for assessment of body composition by DEXA were 0.87% for percentage body fat, 0.53 kg (1.6%) for fat mass, 1.05 kg (2.3%) for lean mass, measured on consecutive days [37].

### 2.6. Blood Sampling

Fasting blood samples were obtained by venepuncture after subjects were seated for more than ten minutes. Plasma from EDTA (Ethylenediaminetetraacetic acid) was stored at −80 °C until analysis. Fasting plasma triglycerides, total cholesterol, HDL and glucose concentrations were determined using a commercial assay kit with a Konelab 20XT clinical chemistry analyser (Thermo Fisher Scientific, Waltham, MA, USA). These tests have good precision and accuracy with coefficient of variation within and between assays (<5.0%). Low density lipoprotein cholesterol (LDL-C) was calculated using the Friedewald equation [38]. Fasting plasma insulin concentrations were determined by ELISA (enzyme-linked immunosorbent assay) (Mercodia, Uppsala, Sweden). This assay has good precision and accuracy with coefficient of variation within and between assays (<4.0%). Measures of insulin sensitivity (homeostatic model assessment (HOMA) of insulin resistance, insulin sensitivity and beta cell function) were calculated from the glucose and insulin levels using the HOMA 2 online calculator [39]. High sensitivity CRP was analysed by were measured using Konelab 20XT clinical chemistry analyzer (range 0.25–40 mg/L, Thermo Scientific, Vantaa, Finland). The coefficient of variation for hs-CRP at median levels (2.4 mg/L) are intra: 0.8%, inter: 1.8%.

### 2.7. Nutrient Intake and Physical Activity Measurements

A set of food scales was provided with instructions on how to accurately weigh (to the nearest gram), measure and record intake in a food diary for 3 weekdays and one weekend day prior to the next visit. Weighed or measured food intake was recorded for 4 days prior to each assessment visit at baseline and weeks 6, 12, 18 and 24. Subjects were instructed to maintain similar patterns of physical activity throughout the study and 4 day physical activity diaries [37] were completed at the same time points as the food diaries. Energy intake and macronutrient profiles were calculated using Foodworks^®^ Nutritional software (version 7, Xyris Software Pty Ltd., Highgate Hill, Queensland, Australia).

### 2.8. Statistical Analysis

All data were checked for errors, hs-CRP levels >10 ml/L were removed from analysis (indication of a possible acute infection). General linear repeated measures analysis of variance was used to compare the mid-point data (energy intake, physical activity energy expenditure and anthropometric measures only) and at the end of each with order included in the analysis and baseline used as a covariate, using SPSS version 21 (SPSS Inc., Chicago, IL, USA). Body composition, anthropometric measures, energy and macronutrient intakes and cardio-metabolic measures were correlated where a relationship was predicted.

## 3. Results

### 3.1. Subjects

**Figure 1:**
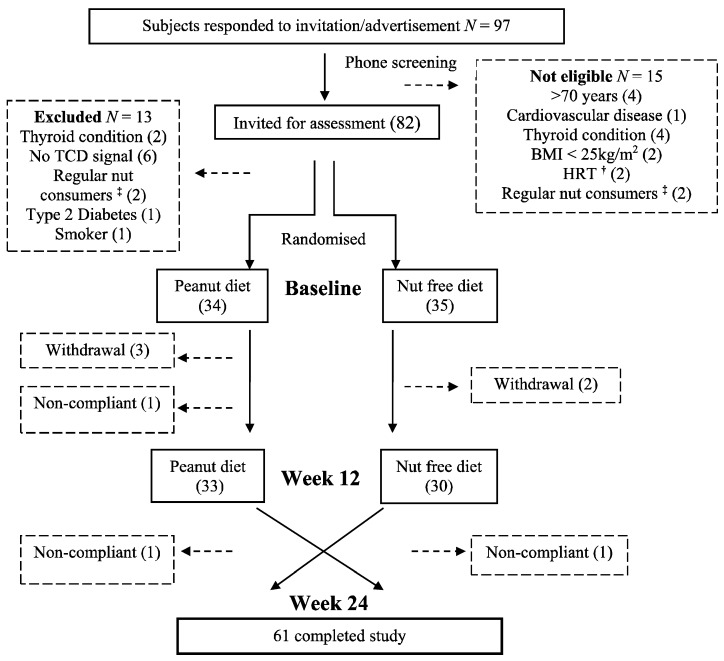
Consort diagram of subjects who were screened, enrolled and completed the study. **^†^** Hormone replacement therapy; **^‡^** consuming ≥30 g nuts/week.

Sixty one subjects (29 males, 32 females) completed the study, with a mean BMI of 31 ± 4 kg/m^2^ and mean age of 65 ± 7 years at baseline. Figure 1 shows a CONSORT diagram of subjects who were screened and enrolled in the study. After commencement of the study 5 subjects withdrew for either health reasons not related to the study or due to time constraints. Three people were excluded for not complying with the study criteria. Compliance with the peanuts, as reported from the diet diaries, was high: 85% (48.7 ± 1.1 g/day) for females and 80% for males (67.2 ± 3.3 g/day). This was confirmed by return of 80% of peanut packets for all subjects.

### 3.2. Nutrient Intake and Energy Balance

Baseline energy and nutrient intakes are presented in Table 2 and Table 3. Analysis of the data revealed that consumption of high oleic peanuts increased energy intake by 10% (853 kJ) compared with the nut free diet (Table 3). The increase could be attributed to a 23 g (~30%) increase in intake of fat which was predominantly monounsaturated fat (22 g increase), with a small significant increase in polyunsaturated fat intake (Table 2). A 12% increase in protein intake and 15% increase in fibre were observed with peanut consumption. No significant changes in carbohydrate or alcohol intake were observed between the two dietary phases (Table 2). No differences were observed in energy expenditure from physical activity diaries between the two dietary phases (Table 3).

### 3.3. Anthropometric Measures and Body Composition

A change in body mass was observed with consumption of high oleic peanuts, with a small significant increase at 6 weeks (0.4 kg) and at 12 weeks (0.5 kg) with peanut consumption compared with the control phase (Table 3). No changes in waist circumference or body composition measures were observed with peanut consumption (Table 3). Calculations of body mass per total daily kJ consumed revealed a difference of −1.0 g/kJ and −1.3 g/kJ at weeks 6 and 12 respectively with peanut consumption compared with control. Monounsaturated fatty acids (MUFA) intake was inversely associated with body fat mass (*r* = −0.264, *p* = 0.042).

**Table 2:** Effect of daily high oleic peanut consumption and nut free diet on macronutrients, fibre and alcohol intakes.

Nutrient Intake	Baseline (SD)	Peanut Diet (SD)	Control Diet (SD)	Difference Peanut-Control (SEM)	*p* Value
Protein (g)	95.8 (31.1)	104.2 (27.4)	95.2 (25.9)	8.9 (2.7)	0.005
Carbohydrate (g)	221.2 (61.9)	202.0 (47.5)	206.4 (52.7)	−4.3 (4.7)	0.630
Total fat (g)	68.3 (23.4)	93.4 (20.7)	71.0 (20.7)	22.7 (2.4)	<0.001
MUFA (g)	25.6 (8.3)	47.5 (11.3)	25.2 (7.7)	22.3 (1.2)	<0.001
PUFA (g)	10.1 (4.1)	11.2 (3.3)	9.8 (3.3)	1.5 (0.4)	<0.001
SFA (g)	26.8 (8.4)	31.8 (8.7)	29.6 (9.7)	2.2 (0.9)	0.141
Alcohol (g)	7.5 (7.1)	8.6 (8.9)	9.6 (10.1)	−1.1 (14.4)	0.454
Fibre (g)	27.2 (7.9)	27.0 (6.9)	23.4 (7.0)	3.5 (0.6)	<0.001

Abbreviation: SD, standard deviation; SEM, standard error of the mean; PUFA, polyunsaturated fat; SFA, saturated fatty acids; MUFA, monounsaturated fat.

**Table 3:** Effect of 6 and 12 week high oleic peanut consumption and nut free diet on energy intake, physical activity energy expenditure and anthropometric and body composition measures.

	Baseline (SD)	6 Week Peanut (SD)	6 Week Control (SD)	Peanut-Control (SEM)	*p* Value	12 Week Peanut (SD)	12 Week Control (SD)	Peanut-Control (SEM)	*p* Value
Daily energy intake (kJ)	8253 (2061)	9004 (2117)	8262 (1925)	1137 (317)	<0.001	9227 (1800)	8292 (1828)	617 (371)	<0.001
Daily PA energy expenditure (kJ)	3801 (807)	3628 (640)	3877 (1128)	−411 (261)	0.520	3879 (965)	3689 (698)	−79 (232)	0.940
Body mass per kJ consumed (g/kJ)	11.4 (4.2)	10.3 (2.6)	11.3 (3.2)	−1.0 (0.3)	<0.001	9.7 (1.9)	11.0 (2.5)	−1.3 (0.3)	<0.001
Body mass (kg)	87.7 (14.1)	88.4 (14.1)	88.0 (14.2)	0.4 (0.2)	0.010	88.3 (14.3)	87.8 (14.5)	0.5 (0.2)	0.010
Waist circumference (cm)	100.0 (11.0)	100.3 (11.2)	100.2 (12.0)	−0.5 (0.3)	0.590	99.8 (11.4)	99.5 (11.9)	0.3 (0.3)	0.780
BMI (kg/m^2^)	30.6 (4.1)	30.9 (4.2)	30.7 (4.1)	0.1 (0.1)	0.050	30.9 (4.2)	30.6 (4.2)	0.2 (0.1)	0.742
% body fat	-	-	-	-	-	38.9 (9.1)	38.4 (9.3)	0.3 (0.2)	0.144
Non bone lean mass (g)	-	-	-	-	-	49174 (1344)	49268 (1392)	−97.1 (160.2)	0.611
Bone mineral content (g)	-	-	-	-	-	2999 (668)	2998 (658)	1.1 (10.3)	0.856

Abbreviation: SD, standard deviation; SEM, standard error of the mean; PA, physical activity; BMI, body mass index.

### 3.4. Fasting Blood Glucose, Insulin, Lipids and C-Reactive Protein

*C*-reactive protein data was analysed in two cohorts: Subjects with baseline CRP levels ≤ 3.5 mmol/L and those with baseline CRP > 3.5 mmol/L (reflecting inflammation). There were no differences in either of these sub-groups between the two treatments. Similarly there were no differences observed in lipids or glucose regulation between treatments (Table 4). An increase in MUFA intake correlated with a decrease in insulin levels (*r* = −0.283, *p* = 0.033).

**Table 4:** Effect of 12 week high oleic peanut consumption and nut free diet on fasting blood lipids, inflammation (*C*-reactive protein) and glucose regulation.

Measure	Baseline (SD)	12 Weeks Peanuts (SD)	12 Weeks Control (SD)	Difference Peanut-Control (SEM)	*p* Value
Total chol. (mmol/L)	5.1 (0.8)	5.2 (0.8)	5.1 (0.8)	0.0 (0.1)	0.662
LDL-C (mmol/L)	3.3 (0.7)	3.6 (0.8)	3.5 (0.8)	0.0 (0.1)	0.421
HDL-C (mmol/L)	1.4 (0.3)	1.4 (0.3)	1.4 (0.4)	0.0 (0.0)	0.190
Triglyceride (mmol/L)	1.2 (0.6)	1.2 (0.7)	1.3 (0.6)	−0.1 (0.1)	0.129
LDL/HDL	2.6 (0.2)	2.6 (0.2)	2.6 (0.2)	0.0 (0.0)	0.819
*C*-reactive protein (mg/L)	1.8 (2.2)	2.1 (1.7)	2.3 (1.9)	−0.3 (0.2)	0.620
Glucose (mmol/L)	5.5 (0.7)	5.5 (0.6)	5.5 (0.5)	0.0 (0.1)	0.614
Insulin (mU/L)	7.9 (4.5)	7.8 (5.4)	7.9 (5.1)	−0.1 (0.5)	0.412
HOMA IR	1.1 (0.6)	1.1 (0.7)	1.1 (0.6)	0.1 (0.1)	0.769
HOMA IS	114.3 (54.5)	124.2 (58.1)	119.9 (56.3)	7.3 (9.0)	0.701
β cell	83.0 (31.7)	80.2 (30.4)	80.4 (32.1)	1.3 (3.9)	0.900

Abbreviation: SD, standard deviation; SEM, standard error of the mean; HDL-C, high density lipoprotein cholesterol; LDL-C, low density lipoprotein cholesterol; HOMA, homeostasis model assessment; IR, insulin resistance; IS, insulin sensitivity.

## 4. Discussion

This is the first study to investigate the effect of Australian high oleic peanut consumption on cardio-metabolic measures, body composition, body mass or waist circumference. In addition, no previous peanut studies have measured inflammatory markers and no intervention studies have measured their effect on glucose regulation. The food diaries revealed ~50–70 g daily intake of peanuts, consumed by both men and women (6 days per week). The expected increase in energy intake from this amount of peanuts was 1400 kJ per day (assuming no other changes were made to the diet). The actual increase in energy intake was ~850 kJ (60% of the expected value) indicating that some of the peanuts were substituted for other foods in the diet. Additional monounsaturated fat consumed during the peanut phase accounted for ~90% of the additional energy consumed. The predicted weight gain for this increase in energy reported was 0.9 kg over 6 weeks and 1.9 kg over 12 weeks (calculated as 1 g body fat mass increase predicted for every additional 37 kJ consumed). The actual average weight gain over the whole period was only 0.5 kg (26% of expected), and no changes in waist circumference or body composition measures were observed. However, it is acknowledged that this predicted weight gain calculation does not take into account the dynamic nature of weight loss hence, may be overestimated.

Energy expenditure from physical activity remained the same for both phases so this did not contribute to the findings. Calculations revealed ~−1.1 g difference in body mass per unit (kJ) of total daily energy intake with peanut consumption compared with the control phase. The body mass per kJ would be expected to be the same for each phase, *i.e.*, as energy changed body weight would expect to change proportionally. This was not observed; for every kJ consumed during the peanut there was less body weight change compared with the control phase. Similarly, eight weeks consumption of 89 g/day of peanuts compared with a nut free diet for 8 weeks by healthy subjects with no dietary compensation demonstrated a weight gain of only 1 kg which was 28% of the predicted 3.6 kg [40]. A possible reason for less than predicted weight gain with additional energy intake was incomplete fat absorption from the peanuts. Fat contained within walled cellular structures of nuts has been found to be incompletely digested in the gut [41] which is possibly compounded by incomplete mastication [42].

In addition the body fat storage may have been limited as a result of the unsaturated fatty acids being oxidized. Unsaturated fats and have a greater thermogenic effect [43] and are more readily oxidised [44] than saturated fatty acids making them less readily stored in the adipose tissue. Further, peanut consumption for 8 weeks has elicited a small (5%) but significant increment in resting energy expenditure in obese individuals [40]. A recent study with high-oleic peanuts has also revealed a greater increase in diet induced thermogenesis compared with conventional peanuts [45] with MUFA intake suggested to contribute to the difference. Other studies have demonstrated an increase in diet induced thermogenesis and fat oxidation with MUFA possibly due to a higher stimulation of the sympathetic nervous system by MUFA than other fatty acids [46].

Interestingly, in this study MUFA intake was inversely associated with body fat mass. It has been suggested that fat quality may have a stronger correlation with weight gain than fat quantity [47]. Studies suggest a role for preferential oxidation and metabolism of dietary MUFA, which influences body composition hence ameliorating the risk of obesity [48,49]. It is possible that a combination of these mechanisms contributed to inefficient energy utilisation or an increase in energy expenditure from thermic effect of consuming peanuts, demonstrating no body composition changes and a less than predicted increase in body weight. This study demonstrates that, despite a smaller than predicted increase in body weight, large doses of peanuts should not be added to the diet but should be substituted for other foods. In a previous study we observed a 10% reduction in energy intake over 4 days when high oleic peanuts were substituted for another snack food (potato crisps), indicating that peanuts have the potential to be included in a weight management diet when substituted for other snack foods [2].

Surprisingly, lipid levels were not altered with high oleic peanut consumption, contrary to many other nut studies [31]. However, the cholesterol lowering effects of nuts are shown to be greatest in individuals with higher baseline LDL and lower BMI [31]. Obese individuals have demonstrated an attenuated cholesterol-lowering response to dietary manipulation of fatty acids compared with lean individuals [50]. The subjects in this study were overweight or obese with an average BMI of 30.6 kg/m^2^ and had on average healthy baseline blood lipid levels. Nut consumption has also been found to lower triglyceride levels predominantly in individuals with hypertriglyceridemia [32]. Similarly, Mukuddem-Peterson *et al.* [51] also reported no changes in blood lipid levels in individuals with obesity with either walnut or cashew consumption. In addition, the lipid lowering effects of nuts are greatest when they are substituted for saturated fat in the diet [31]; in the current study there were no differences in saturated fat intake.

This study demonstrated no differences in glucose or insulin levels with consumption of high oleic peanuts. Similarly several other studies have not shown benefits with consumption of pistachios, almonds and walnuts on fasting glucose or insulin [51,52,53,54,55,56,57,58,59,60,61]. Some short-term intervention studies have shown benefits of nut consumption on glucose homeostasis [22,26,27] and insulin secretion [21,26,27,62,63]. However, when adjustments were made for weight reduction in one of these studies no changes in insulin sensitivity were found [62].

The effects of nuts on insulin sensitivity are influenced strongly by changes in body weight which may have accounted for the changes observed in one of these studies. As there were no changes in body composition in this current study, improvements in glucose regulation were less likely. Improvements in glucose regulation have also been demonstrated when nuts are included as part of an intervention diet such as the Nordiet [21] or a Mediterranean diet [26], with benefits partly attributable to other components of these diets. In the current study an increase in MUFA correlated with a decrease in insulin. A recent review [48] has outlined studies determining the effect of MUFA on insulin resistance, demonstrating improved insulin sensitivity and glucose regulation following MUFA-rich diets in both healthy [64,65,66] and diabetic individuals [67,68,69]. However, these benefits are more likely to be observed in subjects of lower body weight [52,70].

Consumption of high oleic peanuts in the chronic study did not affect the inflammatory marker *C*-reactive protein (CRP) when compared with the nut free diet. Similarly, 12 weeks of hazelnut consumption (60 g) resulted in minimal effect on inflammatory markers and cell adhesion molecules in this group of healthy, normocholesterolemic overweight and obese individuals [71]. In addition, in a recently published review [23], we identified that 50% of nut studies demonstrated no significant differences in inflammatory markers. One of the suggested reasons for this was recruitment of healthy individuals who may only demonstrate limited improvements. Subjects in this study were healthy with a mean baseline CRP of 1.5 mg/L, well below the cut off of 3.0 mg/L for of cardiovascular disease risk [71]. In addition, central adiposity is associated with increased CRP levels and it is possible that those with a central adiposity may not demonstrate improvements in inflammatory markers without weight loss [72]. Both male and female subjects in this study displayed central adiposity. Two other nut studies also demonstrated no change but did observe reductions in other inflammatory markers, IL-6 and VCAM-1 with large doses (65–100 g) of pistachios and walnuts respectively [22,73]. It is possible in the study that other inflammatory markers were improved with high oleic peanut consumption.

A minimum nut dose of 30 g may be required to elicit benefits for inflammatory markers [23]. The current study used a relatively large dose of nuts (~50–70 g), so this was not likely to be a limiting factor. A more likely reason is the population studied; subjects had CRP levels within the normal range for a healthy population at baseline. Despite no observed improvements in cardio-metabolic outcomes, epidemiological studies suggest that prolonged peanut consumption in this population may help to maintain cardio-metabolic health over time [1].

A strength of this study was the randomised, controlled cross-over design which assessed the effects of high oleic peanuts in the same individuals, hence reducing between subject variability. Another strength was high compliance with the consumption of peanuts and with the nut free diet, as assessed by food diary entries and returned peanut packages. The study also controlled for physical activity levels, with subjects requested to maintain similar physical activity levels throughout the study.

One of the limitations of the study was the inability to double-blind the intervention; however, data collection and data entry was blinded to minimise investigator bias. Another limitation was the high dose (actual intake of 50–70 g for both males and females) of peanuts used as proof of concept. A dose of 42 g/day recommended by The American Heart Association for reduction of cardiovascular disease (CVD) may still provide some cardio-metabolic benefits; however, this needs to be confirmed with further investigation. This study did not include a washout period; however, the control phase was a habitual (nut free) diet for 12 weeks, allowing sufficient time for reversal of any effects of peanut consumption [74]. Another limitation was only one blood sample was taken at each intervention period which would not have detected intra-individual variation of lipids.

Due to budget constraints, it was not possible to measure fat excretion and energy expenditure directly, which may have provided information on a possible mechanism for the observed weight maintenance and body fat despite an increase in energy intake. Future studies should consider these outcomes.

## 5. Conclusions

After 12 weeks of incorporating high oleic peanuts into the diet, only a small increase in body weight was observed, despite a large additional amount of energy consumed from the peanuts, with the weight increase being far less than predicted. The dose of ~60 g/day current study was substantial, much greater than the mean nut intake of 5 g/day consumed in Australia [75]. Cardio-metabolic benefits have been observed with 30 g/day of nuts [76] with little impact on body weight [14]. Therefore recommendations can be made to incorporate moderate amounts of high oleic peanuts as part of a healthy diet with unlikely detrimental effects on body weight especially if substituted for other foods. No improvements in cardio-metabolic effects were observed in this healthy population; however it is possible that peanuts have the potential for maintaining cardio-metabolic health or providing cardio-metabolic benefits in at-risk groups.
